# A National Cattle Commission

**Published:** 1881-01

**Authors:** 


A NATIONAL CATTLE COMMISSION.—We give on another page the views expressed by Secretary Sherman in his last annual report, regarding the exportation of American cattle, and the creation of a National Cattle Commission. The facts are briefly these: England is afraid that certain contagious diseases may be carried by American cattle among its own herds. The Privy Council has therefore scheduled against all American cattle, sheep, and hogs brought to English ports. It compels their immediate inspection, the slaughter of all diseased animals at once, and the slaughter of all neat cattle within ten or twelve days after arrival. These regulations cause a loss to American stock dealers of from one-and-a-half to two-and-a-half millions of dollars a year. Now, the actual facts regarding the danger of contagion are, that scarcely any contagious diseases are exported from this country at all. No pleuro-pneumonia has, we are informed, ever been certainly found among American cattle landed in Liverpool. Out of 10,670 cattle, 5,960 sheep, and 2,237 hogs landed from the United States in that port, from June 2 to August 13, there were found but six cattle condemned as having pleuro-pneumonia and three condemned with the foot and mouth disease, ten sheep condemned with scab, and twenty-three hogs with swine fever.

With such statistics as these, it is natural to believe that if the English Government could have some official guaranty that American cattle were free from disease, they would remove their present restrictions against them. The Secretary of the Treasury, therefore, recommends the creation of a Cattle Commission. Its duties are to be to investigate reports of the existence of the various contagious diseases, and to collect information respecting them, reporting the results to some department for official publication. The commission should also be authorized to co-operate with State and municipal authorities, and persons or corporations engaged in the transportation of neat cattle, and to establish regulations regarding them. And, finally, such a commission should be authorized to establish quarantine stations, and to make regulations for such stations, if necessary.

The creation of such a commission would, we believe, be of the greatest value to the live stock interests of the country. The project was endorsed by the convention of live stock owners held at Chicago some time ago. It is greatly to be hoped that Congress will take the desired action in the matter.

